# The Acetobacteraceae: Extending the Spectrum of Human Pathogens

**DOI:** 10.1371/journal.ppat.0020036

**Published:** 2006-04-28

**Authors:** David Fredricks, Lalita Ramakrishnan

Patients with chronic granulomatous disease (CGD) get recurrent infections with a variety of bacterial and fungal pathogens as a consequence of phagocyte defects in production of antimicrobial reactive oxygen metabolites. Patients with CGD often present with clinical syndromes, such as pneumonia or lymphadenitis, for which no credible pathogen is identified, leading to empirical broad-spectrum antibacterial and antifungal therapy. The question beleaguering the clinician in this scenario is whether the patient is infected with a common microbe (e.g., *Aspergillus fumigatus, Nocardia asteroides, Staphylococcus aureus*) that has eluded detection, or a novel fastidious microbe.

In this issue of *PLoS Pathogens,* David Greenberg, Steven Holland, and colleagues [1] have isolated and characterized a new bacterium, *Granulobacter bethesdensis,* from the lymph nodes of a patient with CGD and recurrent idiopathic lymphadenitis. They have shown that *G*. *bethesdensis* represents a new genus and species in the Acetobacteraceae family. The bacterium was repeatedly isolated in charcoal–yeast extract medium from several lymph nodes of the patient over several months. The patient's serum from prior to the current episode had been banked, allowing them to demonstrate seroconversion by whole lysate immunoblot and immunoelectron microscopy. Furthermore, the patient's *G*. *bethesdensis* isolate produced a similar pyogranulomatous lymphadenitis in mouse models of CGD but not in control mice. Finally, the 16S rRNA gene sequence of the bacterium reisolated from infected mouse tissues was identical to the sequence from the bacterium originally isolated from the patient. Do these data prove that *G*. *bethesdensis* is a cause of lymphadenitis in patients with CGD?

Some 124 years after they were proposed, Koch's postulates, so named by the students of Robert Koch, remain the gold standard for proving that a microbe is the cause of a disease. In one of the most influential papers in the history of microbiology, “Die Aetiologie der Tuberkulose” (“The Etiology of Tuberculosis”), presented before the Physiological Society of Berlin in 1882, Koch tried to convince his colleagues that a novel bacterium, *Mycobacterium tuberculosis,* was the cause of tuberculosis [2].

The elements of Koch's postulates are summarized in Box 1, and it is clear that the authors have left no stone unturned to fulfill these postulates to provide a causal link between their new, isolated organism and the CGD episode in this patient. In considering them one by one, it is important to note that elements 1 and 2 were problematic even in Koch's time: he noted difficulties in fitting all microbe–disease associations to his causal paradigm—for instance, in the case of *Vibrio cholerae,* wherein some subjects were found to be colonized with the bacterium without having the characteristic cholera disease [3]. Advances in microbiology since the 19th century have demonstrated the important contributions of the host (immunity), vector, and environment to disease susceptibility and response, and these elements are not considered in Koch's original postulates. Indeed, even in the case of tuberculosis, many asymptomatic individuals harbor *M*. *tuberculosis* for prolonged periods without suffering from the disease. In recent decades, elements 1 and 2 have become ever more complex as the distinction between commensal and pathogen has further blurred as a result of modern medical advances such as cytotoxic cancer therapies and immunosuppression related to transplantation. Increasingly, one person's commensal may be another person's pathogen. For instance, Candida albicans can produce disseminated disease in patients with neutropenia, but it is also a common colonizer of the gastrointestinal tract of immunocompetent humans in whom it does not produce disease. Further complicating matters, not every episode of neutropenic fever is caused by *C*. *albicans,* as other fungi, bacteria, and viruses are also pathogens in this setting. Koch's postulates were conceptualized at a time when our attention was focused on clinical syndromes such as anthrax and pulmonary tuberculosis, which were so distinct that they were easily recognizable even by the laity. Many infectious syndromes in today's practice are without hallmark symptoms and signs. Not surprisingly, then, the authors' attempt to address elements 1 and 2 by examining the reactivity of serum from patients with CGD and from controls to the new bacterium led to ambiguous results: three of 19 patients with CGD versus one of 20 controls were positive. These results are difficult to interpret given that we do not know if the organism is an occasional transient human commensal, and do not know how long immunoreactivity is maintained after exposure. Encouragingly, the authors report the recent isolation of molecularly distinct isolates of G. bethesdensis from two other patients with CGD presenting with a similar syndrome.

The authors have done an outstanding job addressing elements 3 and 4. In this regard, they were tenacious in pursuing different growth media and were fortunate that one of them was hospitable for the pathogen's growth. However, the application of these elements of Koch's postulates can also present practical problems. For instance, not every pathogen is capable of propagation in the laboratory on lifeless (cell-free) medium, and some of these pathogens include *Mycobacterium leprae, Treponema pallidum,* and all viruses. Thus, modifications have been proposed to make Koch's postulates more comprehensive by allowing their application to viruses (Thomas Rivers) and by incorporating immunological criteria (Alfred Evans), epidemiological criteria (Austin Bradford Hill), pathogenicity (Stanley Falkow) [4,5], and nucleic acid sequence information (Fredricks and Relman) [4]. Greenberg at al. have used immunological and sequence evidence to nicely bolster their case for causation.

Bacteria in the Acetobacteraceae family are not common human pathogens, but are commonly found in soil and associated with plants. The acetobacteraceae are known to convert ethanol to acetic acid and are responsible for the conversion of wine to vinegar. Asaia bogorensis is the only other bacterium in this family that has been associated with human disease and isolated from the peritoneal fluid of a patient undergoing peritoneal dialysis [6]. Both A. bogorensis and G. bethesdensis were identified by sequencing their 16S rRNA genes and performing phylogenetic analysis. The increasing use of sequence-based methods for the identification of microbial pathogens in the clinical microbiology laboratory is likely to greatly expand our understanding of the diversity of microbes pathogenic for humans. It will serve the microbiological community well if the discoverers of these novel microbes apply the same rigor toward establishing causality that was demonstrated by Greenberg at al.

Finally, the data presented in the paper raise intriguing questions about pathogenesis and treatment. From which niche did the bacterium infect these patients? Is infection associated with the consumption of certain fruits or vegetables? How did the bacteria get to the lymph nodes? What is the carbon source used by these bacteria in vivo? From the point of view of treatment, it is disturbing to note the high-level resistance of the bacteria to virtually all classes of antibiotics and treatment failure with the two antibiotics (doxycycline and trimethoprim-sulfamethoxazole) to which the bacteria were transiently susceptible upon isolation. This result highlights the grim problem of antibiotic resistance, either intrinsic to the bacterium or induced by antibiotic therapy. The authors propose to obtain a whole genome sequence of the bacterium, which may begin to answer some of these questions.

**Box 1. Koch's Guidelines for Determining if Etiologic Microbes Are the Cause of Disease**

1. Should be found in every case of the disease

2. Should not be found in individuals without disease

3. Should be isolated in pure culture on lifeless media and be capable of causing the characteristic disease anew upon inoculation in a susceptible host

4. Should be reisolated from the susceptible host

## Abbreviation

CGD: chronic granulomatous disease
